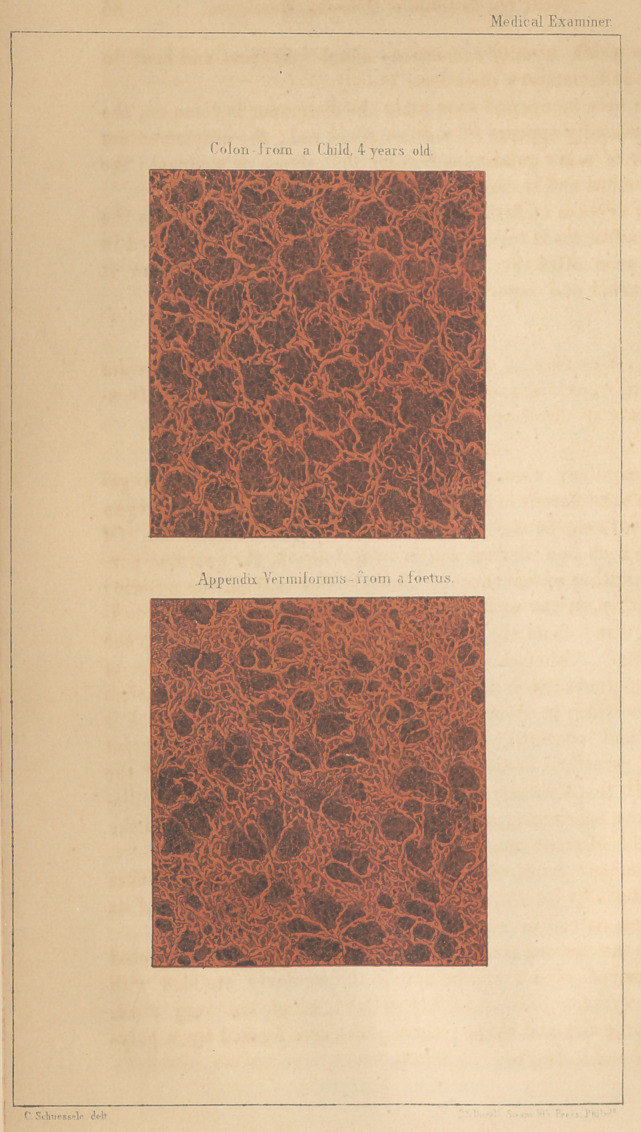# On the Structure of the Mucous Membrane of the Appendix Vermiformis Cœci and Colon

**Published:** 1851-02

**Authors:** John Neill

**Affiliations:** Demonstrator of Anatomy in the University of Pennsylvania


*On the Structure of the Mucous Membrane of the Appendix Vermiformis Cœci and Colon.* By JOHN NEILL, M. D., *Demonstrator of Anatomy in the University of Pennsylvania.*

(With a colored Plate.)

The capillary vessels present great diversities of arrangement in the different organs of the body. Even in the same organ differences exist in their form, interlacement and connection. Of this, we have one illustration in the stomach, the cardiac portion exhibiting an appearance totally different from the pyloric; and another, in the small intestine, the upper portion being easily recognized from the lower, by the forms constituted by the capillaries. Although we cannot always prove a difference of function, where we can show a difference of form, nevertheless it is interesting to observe any peculiarity of structure which is definite and constant; and even if we cannot at present point out any practical benefit resulting from the exhibition of the fact, still, the demonstration of the arrangement of the capillaries in any special organ of the body will have its value to the student of Microscopic Anatomy.

The mucous membrane of the Appendix Vermiformis differs very materially from that of the Colon in the arrangement of its capillaries, as will be seen by the accompanying drawings.

When the mucous membrane of the colon is properly injected and deprived of its epithelium, it is regularly studded with mucous crypts or follicles of Leiberkuhn, which are very much of the same size and shape; the orifices are formed by a polygonal ring of one or two capillaries, which gives the whole surface an appearance very similar to that of ordinary coarse lace. (Fig. 1.) The depth of these crypts is sufficiently great to render the term tubule applicable to them, and unless the epithelium is carefully removed from these tubules by washing, or the preparation dried, so that the epithelium becomes transparent, the capillaries forming the parietes of the crypts or tubules are not evident. The regularity and number of these crypts are sufficient to render the colon distinguishable from the stomach, although numerous high anatomical authorities state that no difference exists between the mucous membranes of these important organs; but when we accurately consider the forms of the orifice, the depth of the crypt, together with the arrangement of the capillaries forming the orifices of the crypts, and the intervening spaces, the difference between the mucous membrane of the stomach and the colon will become evident even to the superficial observer.

The Appendix Vermiformis exhibits a different arrangement of its capillaries from the colon, (Fig. 2.) the crypts are variable in size and shape, and the distances between them are by no means uniform. The interstices between them are formed by a beautiful interlacement of capillaries which presents an extremely regular appearance.

[The same observer has described the anatomy of the mucous membrane of the stomach in the Amer. Jour. of Med. Sciences for Jan. 1851, accompanied by an illustration of the different appearances of that membrane in the cardiac, middle and pyloric portions. The following is his description:

"The surface of the mucous membrane presents different appearances in different portions of the stomach; this fact not having been sufficiently appreciated by observers, we consider as one of the sources of error in the ordinary descriptions of this organ. By far the larger portion exhibits various modifications of the honeycomb structure, the cells are large and polygonal in some parts; in others, they are smaller, deeper, and rounder; the ridges between these cells are formed of one or more convoluted capillaries, and this arrangement of capillaries is particularly evident in the rugæ. The walls of these cells or pockets are formed of a network of capillaries, which subdivides each cell into smaller ones; these cells are what are ordinarily called the orifices of gastric glands, and the subdivision in the bottom of each cell corresponds with the described orifices of tubuli. In the antrum pylori, the structure is modified, the ridges between the cells become larger, more elevated, and as we approach the pyloric orifice, *conical villi* make their appearance; these villi are more numerous and larger towards the pyloric valve, so that fewer of the angular or polygonal cells are visible in their interstices; they are not so large as the villi of the small intestine, but in other respects their external appearances are precisely similar. When well injected, they seem to be composed of capillaries, closely united by a basement membrane, and forming a pyramidal projection.

There may be said to be three different appearanees presented by the microscopic examination of the injected capillaries of the mucous membrane of the stomach when deprived of its epithelium. First. The convexity of a large ruga will have a comparatively smooth and even appearance formed by convoluted and intertwining capillaries. Second. Any other portion excepting the antrum will exhibit cells or alveoli of different sizes and shapes, separated by ridges of various thicknesses, and these ridges are composed of capillaries arranged in the same manner as in the rugæ. Third. In the antrum pylori there are *conical villi*, and cells exist in the interstices and at their bases.

That the main point which we wish to prove may be understood in a few words, we would simply state that we consider the capillaries arranged in the form of 'ridges, cells, and *villi*.' The question may now arise whether this description in any manner deviates from those ordinarily given in the standard works of the day? We consider that it does, particularly with reference to the villi, the existence of which, in the stomach, we wish fully to establish by description and demonstration. It might be asked whether the term *villous* has not consequently been used with reference to the mucous membrane of the stomach? Unquestionably it has, though in a vague and loose way, as indicating a smooth, velvety surface; but not as implying a vascular, papillary projection to which the term *villus* is applied in the intestine."

The variety of opinions that have been expressed in relation to the appearance of the mucous membrane of the stomach has probably arisen from the fact that observers have either failed to inject the mucous membrane, or, what is more likely, have contented themselves with the examination of a *portion* of the stomach only, and have taken that as a type of the whole. Hence we find one author describing it as composed of cells or tubules, and another of villi. To Dr. Neill is due the credit of having first pointed out its varying condition in different portions, and the existence of these "gastric villi."

In regard to the *functions* of these different portions of the stomach, we hazard the opinion that the cardiac portion, where the polygonal cells are most abundant, is mainly intended for the secretion of the *solvent fluid*, the gastric juice; whilst the pyloric portion, in which the *gastric villi* are most abundant, is destined for the *absorption* of such portions of the food as having been reduced to a fluid condition, and possessing the proper degree of tenuity, are enabled to pass into the blood vessels by endosmose; the main object of the villi in this position, being to afford a more extended absorbing surface. That they are not concerned in *chylous* absorption would seem to be proved from their not containing, or communicating with, any chyliferous vessels, from there being no mesentery attached to the stomach, and, of course, no mesenteric ganglia. This view of the function of these portions of the stomach would seem to be supported by the fact that a species of hour-glass contraction of the stomach is sometimes seen to occur, dividing the cardiac from the pyloric portion, the cardiac end retaining the undigested portion, while the fluid materials are passed through the narrowing into the pyloric extremity.* In regard to the question of absorption by the veins of the stomach, it is the opinion of many physiologists, that all thin solutions which require no elaboration, all substances fluid at the temperature of the stomach, and those that are soluble in its secretions, may pass at once into the mass of blood by the physical process of endosmose.† It will at once be seen, therefore, how admirably the villous arrangement of the mucous membrane of the pylorus is adapted to this end by presenting so large an absorbing surface in so small a space.

F. G. S.]

---

*Case of Prolapsus Iridis.* By G. R. Morehouse, M. D., Philadelphia.

James Mahoney, æt. 19, on the morning of Dec. 4th, while engaged at his occupation of sail making, received a severe injury of the cornea in the following manner. In attempting to pass his needle through a tense seam of sail-cloth, it slipped from his grasp and was thrown by the recoil of the seam against the cornea of the right eye, penetrating it midway between its

*Muller's Phys. Vol. I. pp. 550. Beaumont, p. 113. Carpenter's Principles of Physiology, p. 490. Magendie, p. 290.

†Blondlot, Traité Analytique de la digestion.

## Figures and Tables

**Figure f1:**